# Rupture of the intra-articular portion of the long head of the biceps associated with a symptomatic os acromiale

**DOI:** 10.4103/0973-6042.42578

**Published:** 2008

**Authors:** Paul R. Harnett, Philip M. Ahrens, Sri M. Gadikoppula

**Affiliations:** Department of Orthopaedics, Royal Free Hampstead NHS, London, UK

**Keywords:** Internal fixation, long head of the biceps, os acromiale, tenotomy

## Abstract

We report the case of a 45-year-old male who presented with a 5-year history of shoulder pain following an injury. Clinical and radiological investigations revealed a ruptured long head of the biceps and a meso-os acromiale. We performed an arthroscopic resection of the intra-articular stump of the long head of the biceps, followed by internal fixation of the mobile os acromiale using a tension band technique. Rupture of the long head of the biceps associated with a symptomatic os acromiale has not been previously described. This case reinforces the importance of routine shoulder arthroscopy in the treatment of symptomatic os acromiale.

## INTRODUCTION

Os acromiale is an unfused epiphysis of the anterior part of the acromion in a mature individual.[1] It is present in approximately 1.4–8% of the population.[2] Os acromiale has been described in association with the impingement syndrome and rotator cuff tears.

To the best of the authors' knowledge a case of a symptomatic os acromiale associated with rupture of the long head of biceps (LHB) has not been reported in the literature to date. We describe the appropriate treatment and discuss the likely mechanism of the lesion and its clinical relevance.

## CASE REPORT

A 45-year-old right-handed gentleman presented with a 5-year history of chronic left shoulder pain. The symptoms had begun after a fall on ice, with the patient landing directly on his shoulder. At the initial consultation the patient presented with pain over the anterolateral and superior aspect of the shoulder that was exacerbated by overhead activities. He scored 6/10 on a pain visual analogue scale and 4/12 on the Simple Shoulder Test.[3] An x-ray of the shoulder had been performed 2 years earlier and had been reported as normal.

**Figure 1:**
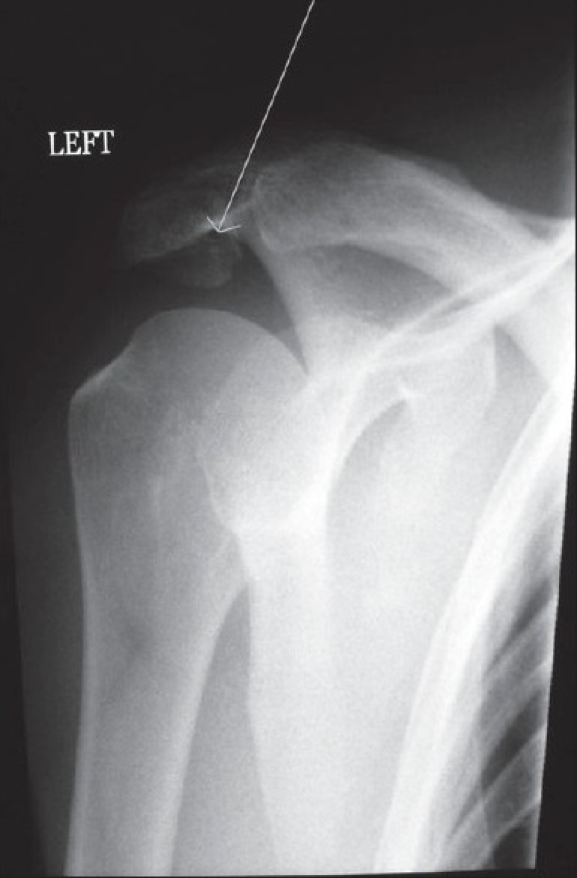
Preoperative AP shoulder radiograph; the arrow is pointing to the os acromiale

**Figure 2:**
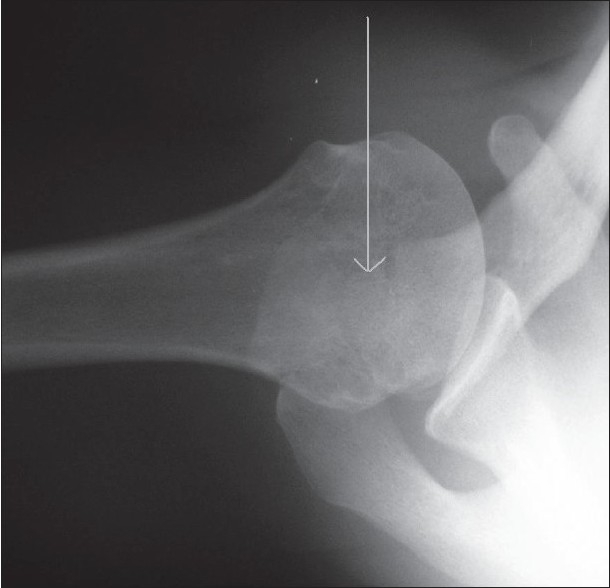
Preoperative axillary shoulder radiograph; the arrow is pointing to the os acromiale

**Figure 3:**
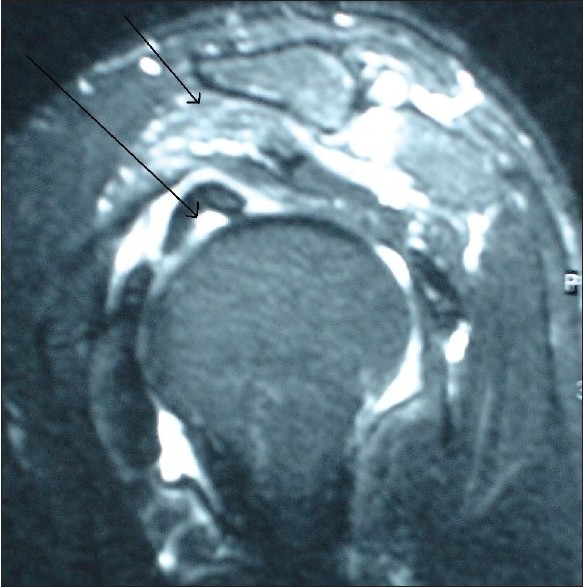
Shoulder MRI. The left arrow demonstartes the intra-articular LHB stump. The right arrow is pointing to the os acromiale

On clinical examination there was reduced range of motion, with active forward elevation up to100° (passive 110°), active abduction up to 120° (passive 130°), external rotation up to 35°, and functional internal rotation to the level of L5. The acromion was tender and mobile; there was tenderness also on palpation of the bicipital groove. There was clinical evidence of a ruptured LHB. Radiological investigations confirmed the presence of an os acromiale, with AP and axillary views demonstrating the unfused epiphysis [Figures 1 and 2]. MRI demonstrated the meso-os acromiale, with edema adjacent to the apophysis, and the intra-articular stump of the ruptured LHB [Figure 3].

### Surgical treatment

**Figure 4:**
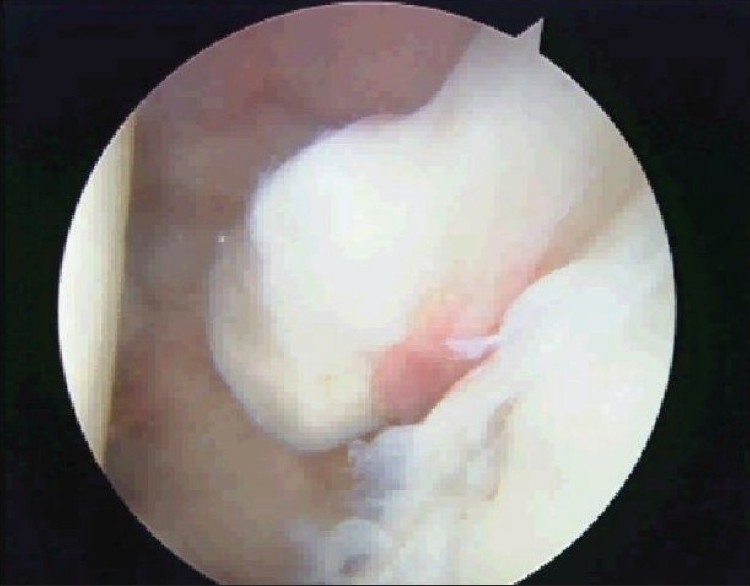
Arthroscopic view of the stump of the LHB before it was removed

Shoulder arthroscopy revealed a 1.5-cm long, hypertrophic, and mobile LHB stump. There was also synovitis of the rotator interval and fraying of the anterior labrum. Excision of the stump was performed using the technique described for a tenotomy of a hypertrophic LHB tendon.[4] The arthroscopic view of the LHB stump is shown in Figure 4.

After completion of the arthroscopy, a limited anterosuperior approach was made, elevating the deltoid at the level of the unfused physis. The cartilaginous apophysis was excised, exposing cancellous bone anteriorly and posteriorly. The bone ends were compressed and fixed with two Kirschner wires and a figure-of-eight tension band technique. The deltoid was repaired with sutures.

**Figure 5:**
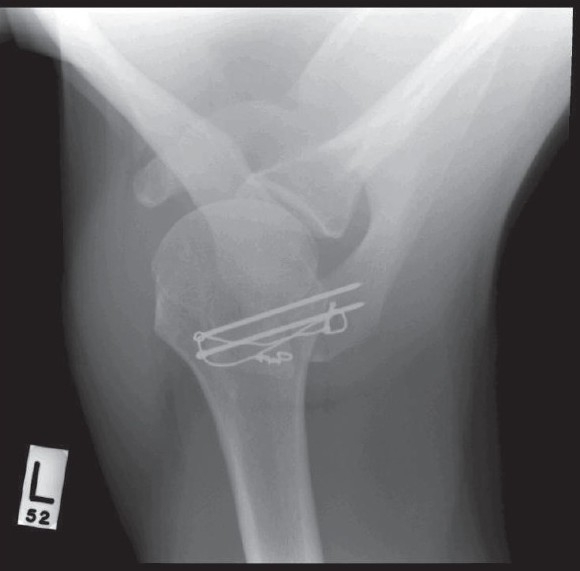
Postoperative x-ray demonstrating the tension band wires holding the fixation

Union was achieved by 9 weeks postoperatively. Postoperative x-ray demonstrated the tension band wires holding the fixation [Figure 5]. At the latest follow-up (at 6 months postoperatively) the patient had full range of motion, he scored 10/12 on the Simple Shoulder Test questionnaire and he was satisfied with the outcome, except for some local discomfort over the wires. He is due for removal of his metalwork.

## DISCUSSION

When conservative measures for symptomatic os acromiale fail to relieve symptoms, surgical intervention should be considered. Options include excision of a meta-acromion, open reduction and internal fixation of meso- and pre-acromia, and arthroscopic decompression.[2]

Literature review showed that trauma was associated with 42% of the cases of os acromiale that required internal fixation.[5] The present case was also associated with a traumatic event.

Os acromiale is a recognised associated finding in patients with subacromial impingement and rotator cuff tears.[6] Rotator cuff tears have been associated with symptomatic os acromiale in over 50% of patients requiring internal fixation of their os acromiale.[5,6]

In our patient, bone grafting was not necessary. Internal fixation without bone grafting has shown good results, with satisfaction rates being as high as 92%.[6]

The association of an os acromiale with a ruptured LHB has not previously been described. In this case, the patient presented with the clinical features of a symptomatic os acromiale and LHB pathology. We cannot be certain when the tendon rupture occurred, although its location at the entrance of the bicipital groove as well as the presence of tendon hypertrophy are suggestive of a rupture caused by persistent impingement and degenerative attrition. It is uncommon for a ruptured LHB to leave a significant intra-articular stump; in this case it was evidently the source of his ongoing symptoms.

Long head of the biceps tenotomy, with excision of its intra-articular portion, has been described for hypertrophic LHB tendons[4] and this was done in the present case to prevent ongoing intra-articular symptoms once the os acromiale had united.

Awareness of this association is required to prevent failures of surgical treatment for os acromiale. MRI in this case demonstrated the LHB pathology; a simple ultrasound may have been difficult to interpret. In cases with an intact rotator cuff on imaging studies, arthroscopy may not routinely be performed. We suggest that diagnostic arthroscopy be performed in all cases scheduled for surgical treatment for os acromiale.
